# Two sides of the same coin: Meta-analysis uncovered the potential benefits and risks of traditional fermented foods at a large geographical scale

**DOI:** 10.3389/fmicb.2022.1045096

**Published:** 2022-11-03

**Authors:** Meng Xu, Shunyong Su, Zeng Zhang, Shuaiming Jiang, Jiachao Zhang, Yanqing Xu, Xiaosong Hu

**Affiliations:** ^1^School of Food Science and Engineering, Hainan University, Haikou, China; ^2^School of Public Administration, Hainan University, Haikou, China; ^3^Key Laboratory of Food Nutrition and Functional Food of Hainan Province, Haikou, China; ^4^College of Food Science and Nutritional Engineering, China Agricultural University, Beijing, China

**Keywords:** traditional fermented foods, shotgun metagenomics, beneficial microorganism, opportunistic pathogen, antibiotic resistance gene

## Abstract

Traditional fermented foods, which are well-known microbial resources, are also bright national cultural inheritances. Recently, traditional fermented foods have received great attention due to their potential probiotic properties. Based on shotgun metagenomic sequencing data, we analyzed the microbial diversity, taxonomic composition, metabolic pathways, and the potential benefits and risks of fermented foods through a meta-analysis including 179 selected samples, as well as our own sequencing data collected from Hainan Province, China. As expected, raw materials, regions (differentiated by climatic zones), and substrates were the main driving forces for the microbial diversity and taxonomic composition of traditional fermented foods. Interestingly, a higher content of beneficial bacteria but a low biomass of opportunistic pathogens and antibiotic resistance genes were observed in the fermented dairy products, indicating that fermented dairy products are the most beneficial and reliable fermented foods. In contrast, despite the high microbial diversity found in the fermented soy products, their consumption risk was still high due to the enrichment of opportunistic pathogens and transferable antibiotic resistance genes. Overall, we provided the most comprehensive assessment of the microbiome of fermented food to date and generated a new view of its potential benefits and risks related to human health.

## Introduction

Fermentation is one of the oldest and most economical methods for food storage and processing in the world. It has been widely used to improve food safety, shelf life, and sensory and nutritional properties (Gao et al., 2021). The International Scientific Association of Probiotics and Prebiotics defines fermented foods as “foods made through desired microbial growth and enzymatic conversions of food components” (Marco et al., 2021). Since the Neolithic Age, fermented foods have been produced and consumed as an integral part of the human diet and cultural traditions (Liu et al., 2018; Anyogu et al., 2021). Fermented foods have become very popular in recent years, mainly because they are considered to be beneficial to health. Studies have found that fermented foods have beneficial effects on human health by promoting antioxidation processes, lowering blood lipids, improving immunity, inhibiting tumors, delaying aging, and preventing gastrointestinal diseases (Marco et al., 2017; Melini et al., 2019; Şanlier et al., 2019). However, in most natural fermentation processes, the microbial community is complex, and the fermentation process is difficult to control, which brings many hidden dangers to fermented foods (Anal, 2019). Food safety incidents caused by the consumption of fermented foods occur from time to time, which is not only unfavorable to the development of the fermented food industry but also threatens the safety of consumers (Anal et al., 2020; Wang et al., 2021). Although some literature has reported the health benefits or safety risks of fermented foods, there has been little comprehensive analysis of the potential health benefits and safety risks of fermented foods (Anal, 2019; Dimidi et al., 2019; Şanlier et al., 2019).

Traditional fermented foods usually depend on natural wild colony fermentation as the main production mode. In fermentation, a variety of microorganisms from raw materials and the environment are usually introduced. This process can involve the use of nutrients in the raw materials to change the texture and edible quality of the food through metabolic pathways and produce a unique fermentation fragrance (Kim et al., 2016; Hwang et al., 2017; Licandro et al., 2020). The microbial ecosystem of fermented foods is an important symbol of its typicality, and it is also the decisive factor in the assessment of the function and risk of fermented foods (Tamang et al., 2016). On the one hand, fermented foods contain microorganisms, which are beneficial to consumption. Some microbes in fermented foods can reach the gastrointestinal tract. Although they do not stay in the intestinal tract for a long time, it is sufficient to enable physiological benefits such as the inhibition of intestinal pathogens and the mediation of epithelial regulation and immune regulation in the intestinal tract (Lebeer et al., 2008; David et al., 2014; Dimidi et al., 2019). On the other hand, the natural fermentation process is often accompanied by the introduction of spoilage bacteria or pathogenic bacteria, which will seriously affect the sensory quality, flavor, and edible safety of traditional fermented foods (Anal et al., 2020; Wang et al., 2021). Therefore, it is necessary and urgent to deeply understand the complex microbiome of traditional fermented foods.

Next-generation sequencing technology has completely changed microbial research, including that regarding the microbial community in food, by enabling high-throughput gene analysis of mixed microbial communities (Consortium, 2012; Fierer et al., 2012). Metagenomics, including two different culture-independent sequencing methods, namely, amplicon sequencing, and shotgun genomics, involves the analysis of genomic deoxyribonucleic acid (DNA) isolated from the whole microbial community (Ranjan et al., 2016). To date, most studies related to fermented foods have employed amplicon sequencing to study the composition of bacteria and fungi, which is usually limited by the identification of the genus level (Leech et al., 2020). Shotgun metagenomics has higher resolution, but due to the high sequencing cost, the application of this method in traditional fermented food microbiology research is still lacking.

Here, based on the shotgun metagenome approach, we explored the microbiome of different traditional fermented foods at the species level; revealed the microbial diversity, taxonomic composition, and metabolic pathways of different fermented foods; and comprehensively analyzed the potential benefits and safety risks of the different fermented foods to human health. We hope to provide a scientific basis for consumers to know more about fermented food and provide direction for the development of the traditional fermented food industry.

## Materials and methods

### Sample collection

**FIGURE 1:**
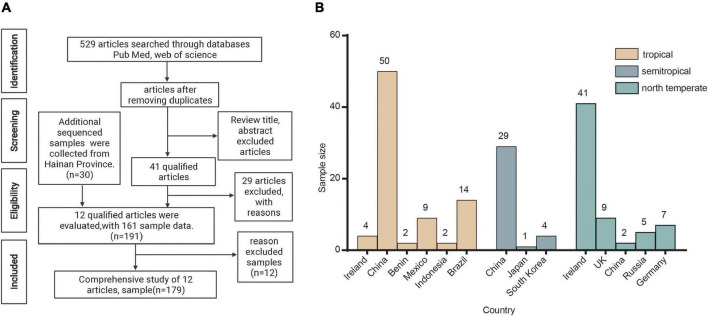
Acquisition of samples. **(A)** Flowchart for the identification of studies and samples. **(B)** Geographical distribution of samples.

A detailed flowchart of the sample collection process is shown in Figure 1. The PubMed/Medline and Web of Science bibliometrics databases were used to systematically search peer-reviewed literature, and the retrieved full text and other reviewed references were used for manual search. We used the following keywords in our search: (“fermented food”) AND (metagenomic OR metagenome OR microbiota OR shotgun). We found 529 references, 463 of which were retained after deduplication; 422 of these were then excluded from the review of the title and abstracts. Of the 41 reserved articles, 29 were excluded because of the use of 16S gene sequencing technology. Finally, excluding the 12 samples whose data could not be obtained in the NCBI databases, 12 references (including 161 samples) were found to have used shotgun metagenomic sequencing technology to study the microbiome of fermented food. In addition, we also collected 30 samples of traditional fermented foods from Wenchang, Qionghai, and Lingshui Li Autonomous Region in Hainan Province. The metadata for the samples are presented in Supplementary Table 1.

### Deoxyribonucleic acid extraction and shotgun metagenomic sequencing

Samples were stored at –20°C until DNA extraction. After homogeneously mixing 10 g of the fermented food sample with 90 mL of sterile NaCl solution (0.85%, w/v), total DNA was extracted from the diluent of the mixture by a QIAamp^®^ DNA Mini Kit (Qiagen, Hilden, Germany) according to the instruction manual. After detection for integrity and purity, the shotgun metagenome sequencing was carried out by an Illumina HiSeq 2500 instrument (Novogene, Beijing, China). The whole metagenome library preparation and sequencing process used services from Beijing Novogene Technology Co., Ltd.

### Data processing and statistical analysis

MetaPhlan 2 software was used to annotate and classify metagenomic species, and HumanN 2 was used to annotate metagenomic metabolic pathways. By comparing the microbial sequences with the CARD database, the abundance of microbial antibiotic resistance genes (ARGs) was obtained (Hitch et al., 2018). Statistical analysis was performed with the Wilcoxon rank-sum test and Kruskal–Wallis test using R and GraphPad. A *p*-value < 0.05 was considered to be significantly different.

## Results

### Literature search and characteristics of included samples

The metadata of the samples are listed in Supplementary Table 1. A total of 179 samples were included in this study, of which 149 samples were taken from 12 published articles (Walsh et al., 2018; Kumar et al., 2019; Sirén et al., 2019; Chacón-Vargas et al., 2020; Leech et al., 2020; Li et al., 2020; Yulandi et al., 2020; de C. Lima et al., 2021; Hu et al., 2021; Liu et al., 2021; Zhang et al., 2021; Tamang et al., 2022) and 30 samples were taken from Hainan Province and sequenced. The samples were classified according to the fermentation raw materials, fermentation substrates, and climatic zone in different regions.

### The microbial diversity of fermented foods classified by different raw materials, substrates, and climatic conditions was different

**FIGURE 2:**
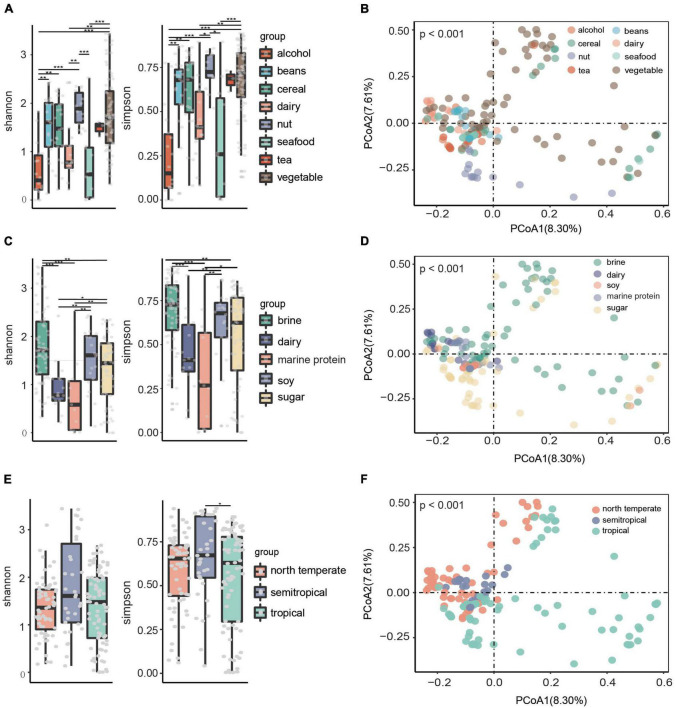
Microbial diversity of different fermented foods. **(A)** Microbial alpha diversity in fermented foods with different raw materials. **(B)** Principal component analysis (PCoA) score plot based on Bray–Curtis distance for all samples in fermented food with different raw materials. **(C)** Microbial alpha diversity in fermented foods with different substrates. **(D)** PCoA score plot in fermented food with different raw materials. **(E)** Microbial alpha diversity of fermented foods in different climatic zones. **(F)** PCoA score plot in fermented food with different raw materials. Each point represents the composition of the microbiota of a sample.

The Shannon and Simpson indices were calculated based on relative abundance at the species level to compare microbial alpha diversity in fermented food samples from different raw materials, substrates, and climatic zones. Principal coordinate analysis (PCoA) based on Bray–Curtis distance was used to compare β diversity. The results showed that there were significant differences (*p* < 0.05) in the microbial α diversity of fermented foods with different raw materials (Figure 2A). The fermented food with dairy and seafood as raw materials had low alpha diversity, while the fermented food with nuts and vegetables as raw materials had high alpha diversity (Figure 2A). The clusters of points representing fermented foods with different raw materials were obviously different in PCoA, which indicates that there were obvious differences in their species structure (Figure 2B). Consistently, fermented foods with dairy and marine protein as substrates had low microbial alpha diversity (Figure 2C), and fermented foods with different substrates had significantly different microbial beta diversity (*p* < 0.05) (Figure 2D). In addition, we found that the α diversity of fermented foods in semitropical regions was significantly higher than that in tropical regions (Figure 2E), and β diversity showed aggregation in different climatic zones (Figure 2F).

### Fermented foods with different substrates differ greatly in microbial composition

**FIGURE 3:**
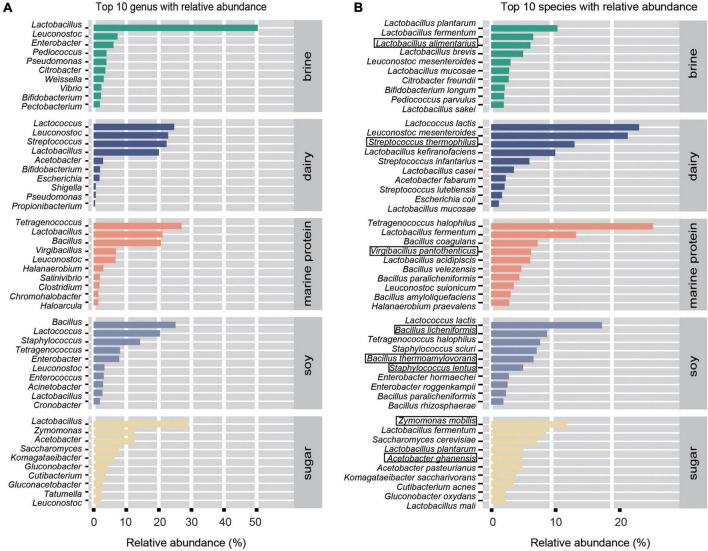
Main (core) microbial composition of fermented foods with different substrates. **(A)** Top 10 genera by relative abundance. **(B)** Top 10 species by relative abundance. The bacteria marked by black boxes are the characteristic bacteria.

**FIGURE 4:**
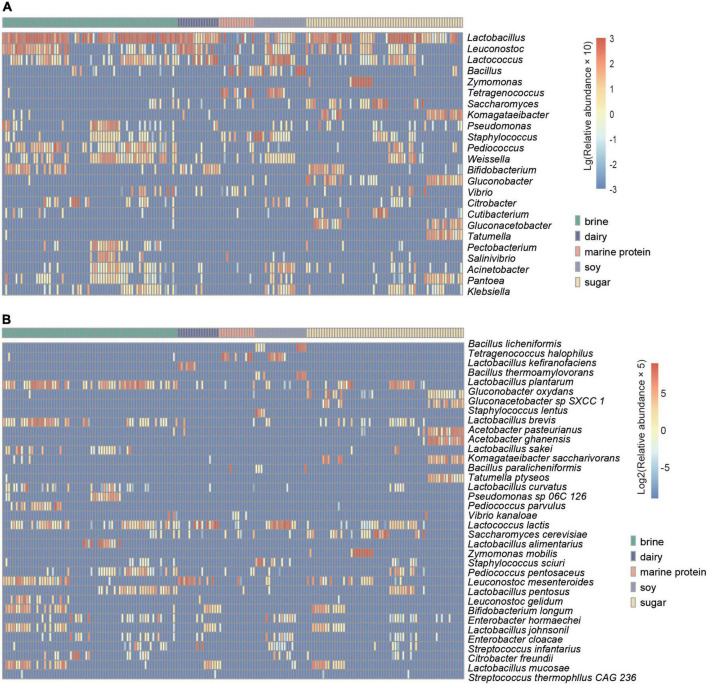
Heatmap showing the distribution of the core differential microbes. **(A)** Variations in significantly differentially abundant genera in different substrate-fermented foods screened by Wilcoxon rank-sum test. **(B)** Variations in significantly differentially abundant species in different fermented food substrates screened by the Wilcoxon rank-sum test. The depth degree of color represents the relative abundance (blue indicates a small number, and red indicates a large number).

To enable the study of more fermented foods, we selected fermented foods with different substrates for more in-depth analysis. Based on the MetaPhlan2 annotation results, as shown in Figures 3A,B, we displayed the top ten bacteria with the highest average relative abundance of microbes at the genus level and species level in different substrate fermented foods and defined these bacteria as core microbes. In addition, the heatmap demonstrated differential microbes with significant differences in relative abundance among the different types of fermented foods (kw test, *p* < 0.05) and mean relative abundance greater than 0.05% (Figures 4A,B). We found that the types of core microbes in fermented foods from different substrates were quite different, both at the genus level and the species level. In addition, some fermented foods had characteristic species, i.e., the core species in a certain substrate fermented food, that were not present in other types of fermented foods. The bacteria marked with a black box in Figure 3B were the characteristic bacteria of various fermented food substrates.

### Analysis of correlation between core microbes and key metabolic pathways

**FIGURE 5:**
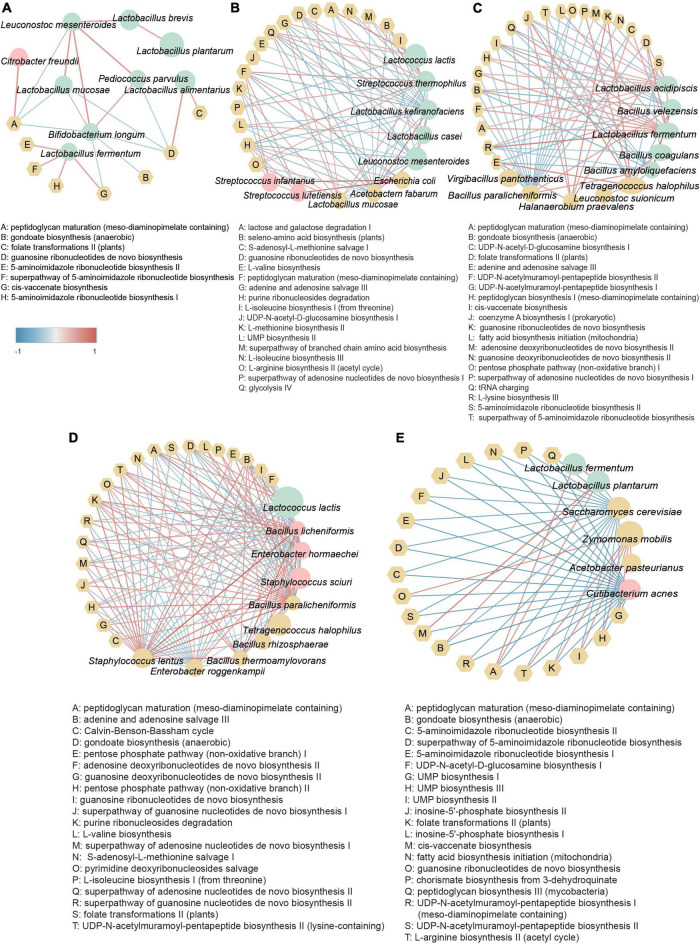
Analysis of correlation between core bacterial species and key metabolic pathways in fermented foods with **(A)** brine, **(B)** dairy, **(C)** marine protein, **(D)** soy, and **(E)** sugar. Correlation analysis was performed using the Spearman method. Data with an absolute value of r greater than 0.4 were selected. The edge width and color (red: positive; blue: negative) are proportional to the correlation strength. The size of that node is directly proportional to the average abundance of the respective population. Pink nodes represent opportunistic pathogens, green nodes represent beneficial bacteria, yellow round nodes represent other types of bacteria, and yellow hexagonal nodes represent metabolic pathways.

To better understand the important roles of microbes in fermented foods, we investigated the associations of core microbes with key metabolic pathways (the top 20 metabolic pathways in relative abundance) in different substrate fermented foods by calculating the Spearman rank correlation coefficient. Network diagrams with correlation coefficients r greater than 0.4 between core microbes and metabolic pathways of various basal fermented foods are shown in Figure 5. The key metabolic pathways of different substrate-fermented foods were significantly different (*p* < 0.05) (Supplementary Table 2). The results showed that metabolic pathways related to cellular structure, replication, and translation were prevalent in fermented foods.

### Potential benefits and risk analysis of fermented food

**FIGURE 6:**
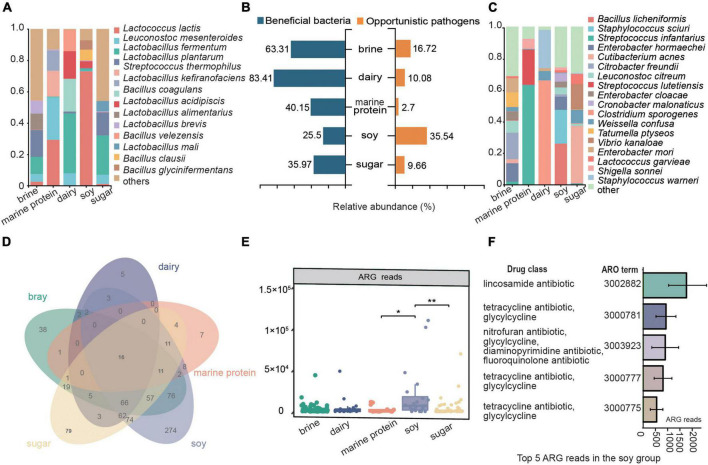
Potential benefits and risk analysis of fermented food. **(A)** Composition of beneficial microorganisms in different substrate fermented foods. **(B)** Enrichment of beneficial bacteria and opportunistic pathogens. **(C)** Composition of opportunistic pathogens in different substrate-fermented foods. **(D)** Venn diagram showing the species distribution of antibiotic resistance genes (ARGs) in different fermented foods. **(E)** ARG reads in different fermented foods. **(F)** The top five enriched ARGs and their corresponding antibiotics in fermented soy foods. ARO term, a taxonomic unit in the CARD database.

To comprehensively analyze the potential benefits and risks of fermented foods to human health, we assessed the beneficial microbes and opportunistic pathogens in fermented foods through extensive literature searches and analyzed the resistance genes of microbes (Figure 6). As shown in Figure 6B, dairy fermented food had the highest abundance of beneficial bacteria, reaching 83.41%, followed by brine (63.31%), and the lowest abundance of beneficial bacteria was found in fermented soy products (25.5%). The relative abundance of opportunistic pathogens was the lowest in marine protein base fermented foods (10.08%), while the most opportunistic pathogens were found in fermented soy products (35.97%), which exceeded the number of beneficial bacteria in fermented soy products. The compositions of various beneficial bacteria and opportunistic pathogens in different fermented foods are shown in Figures 6A,C, respectively. We found that *Leuconostoc mesenteroides* widely existed in other kinds of fermented foods, except fermented soy products. *Lactococcus lactis* was the beneficial bacterium in fermented bean products, and its number accounted for more than 70% of the beneficial microbes in this kind of fermented food. The main opportunistic pathogen in fermented dairy products was *Bacillus amyloliquefaciens*, and *Streptococcus infantarius* was the most important opportunistic pathogen in fermented foods with marine protein. In addition, microbes in fermented soy products had the most types of resistance genes (Figure 6D), and the number of antibiotic resistance gene (ARG) reads was the highest (Figure 6E). The main resistance genes in fermented soy products were lincosamide antibiotic, tetracycline antibiotic, glycylcycline, nitrofuran antibiotic, diaminopyrimidine antibiotic, fluoroquinolone antibiotic, and tetracycline antibiotic resistance genes (Figure 6F). The major resistance genes in other fermented foods were shown in Supplementary Figure 1.

## Discussion

Food fermentation is a valuable global cultural heritage, a rich and valuable microbial resource, but it is also the key to the economy of many regions (Chen et al., 2017). Exploring the microbiome in fermented foods is beneficial to gain a deep understanding of the commonalities and differences of different fermented foods, to better utilize the beneficial properties and to provide theoretical and data foundations for their effects on human health (Leech et al., 2020; Zhang et al., 2021). The high taxonomic resolution of shotgun metagenome analysis has led to more in-depth microbiome identification of fermented foods, which helps us better understand the properties and functions of different fermented foods.

Environmental heterogeneity has always been considered one of the reasons for the formation of microbial diversity patterns (Yang and Wu, 2020). The non-random distribution of microorganisms is affected by environmental factors and is also related to geographical distance (Xia et al., 2016; Zeng et al., 2019). The regional trend found in our study was based on large-scale data covering tropical, subtropical, and north-temperate regions. The result indicated that optimum conditions for the highest bacterial diversity in our studied areas were located at the semitropical zone (Figures 2E,F). Fermentation raw materials and fermentation substrates are important driving factors affecting the microbial composition and metabolic function of fermented foods, and the microbial diversity of fermented foods is largely determined by the types of nutrients available to microbes (Leech et al., 2020; Moonga et al., 2021). Consistently, significant differences in the microbial diversity of fermented foods with different raw materials and substrates were found in our study (Figure 2). Furthermore, more than 5,000 fermented foods are consumed around the world (Leech et al., 2020). To make the results of our study more likely to be applied to more fermented foods, we conducted an in-depth analysis of the microbiome of fermented foods under different substrate categories. We found that metabolic pathways related to cellular structure formation, replication, and translation were enriched in all types of fermented foods, implying that microbial metabolism in fermented foods was robust (Figure 5). Notably, *Lactococcus lactis* and *Lactobacillus casei*, as core microbes in fermented dairy products, were positively associated with the lactose and galactose catabolism pathways, which might explain the response of people with lactose intolerance to fermented dairy products (Figure 5B).

Fermented foods have become the focus of increasing interest as a range of foods claimed to have health benefits. The potential health benefits of fermented foods include a reduced risk of high blood pressure, diabetes, obesity, high cholesterol, diarrhea, blood clots, and more (Marco et al., 2017; Melini et al., 2019). One explanation for the health benefits of fermented foods is related to the beneficial microbes in fermented foods (Orisakwe et al., 2020). In fact, it has been proposed that fermented foods provide a means of safe microbial exposure to compensate for the absence/removal of desirable host microbes to address the “industrialization” of the human microbiota (Chilton et al., 2015; Sonnenburg and Sonnenburg, 2019). *Lactic acid bacteria* (LAB) are an important class of probiotics in the human body, with immunomodulatory, anti-inflammatory, antioxidative, and antiproliferative functions (Matsuzaki et al., 2017; Xie et al., 2019). LAB can survive and multiply in the human intestinal tract, and their growth and metabolism processes not only maintain their own needs but also help to maintain the health of the host organism (Zhang et al., 2019). *Lactococcus lactis* is the most representative strain of lactic acid bacteria, which is considered a safe microorganism by the US Food and Drug Administration and is commonly used in food fermentation, drug production, and feed addition (Mao et al., 2016). Agarwal and Bhasin (2002) found that *Leuconostoc mesenteroides* was beneficial for shortening the duration of diarrhea. *Lactobacillus fermentum* has a clear role in improving liver steatosis, which has been confirmed in some studies (Rivero-Gutiérrez et al., 2017; Nan et al., 2018). As one of the natural flora of the human digestive tract, *Lactobacillus plantarum* has many probiotic functions, including improving intestinal flora, reducing serum cholesterol content, enhancing immunity, discharging heavy metals, and promoting nutrient absorption (Siezen and van Hylckama Vlieg, 2011). In our study, we found that beneficial bacterial resources were abundant in fermented foods and that LAB were enriched in various fermented foods (Figures 6A,B). *Leuconostoc mesenteroides* was widely enriched in fermented foods, except soy fermented foods. *Lactobacillus fermentum* was the main beneficial bacteria in fermented brine, dairy, and marine protein. *Lactococcus lactis* was found to be highly enriched in fermented dairy products and bean products. Moreover, we found that *Lactobacillus kefiranofaciens* was a characteristic bacterium of fermented dairy products, and it has been found that *Lactobacillus kefiranofaciens* has metabolism regulating, immune regulating, and antioxidant functions and improves diabetes, allergies, and brain health (Slattery et al., 2019). *Lactobacillus alimentarius* was abundantly enriched only in the fermented food products using the brine substrate. In addition, the proportion of beneficial bacteria in fermented dairy products was the highest, indicating that fermented dairy products might become the most promising probiotic resource. Thus, our study indicated the enrichment of beneficial bacteria in different fermented foods and provided data support for better utilization of microbial resources in fermented foods.

Unfortunately, the safety of traditional fermented foods has been questioned due to the contamination of pathogenic bacteria and microbes carrying ARGs. The number and types of opportunistic pathogens in different fermented foods were inconsistent (Figure 6). The marine protein-based fermented food had the least opportunistic pathogens (5.92%), followed by fermented dairy products (10.08%), and fermented soybean products (33.63%) had the highest enrichment in opportunistic pathogens, which seemed to indicate that fermented soy products carry a higher potential risk for disease. *Staphylococcus sciuri*, *Enterobacter hormaechei*, and *Bacillus licheniformis* were the core species of fermented soy products and the main opportunistic pathogens (Figures 3B, 6C). *Staphylococcus sciuri* is a human pathogen that causes a wide range of human infections, such as endocarditis, peritonitis, septic shock, urinary tract infection, pelvic inflammatory disease, and wound infection (Li et al., 2011). *Enterobacter hormaechei* causes urinary tract infection, hemorrhagic diarrhea, septicemia, and bloodstream infection (Campos et al., 2007; Kumar and Das, 2016; Parra-Flores et al., 2018). *Bacillus licheniformis*, a characteristic bacterium of fermented soy products, is considered a pathogen causing infection in humans and can cause skin infection and sepsis (Ameur et al., 2005; Matua et al., 2015). The main conditional pathogen in fermented marine protein products was *Streptococcus infantarius*, which can cause biliary tract infection, endocarditis, colorectal cancer, and hepatobiliary diseases (García-Garrote et al., 2014; Kaindi et al., 2018). *Citrobacter freundii* can cause skin, lung, and systemic diseases (Nakazawa et al., 1983; Chuang et al., 1998) and is enriched in brine-fermented food. *Cutibacterium acnes* was considered to be the main cause of prosthetic joint infection after shoulder arthroplasty (Carabeo, 2011) and was enriched in fermented food based on sugar. We found that *Weissella confusa*, which causes disease in immunocompromised humans and animals, was in content in fermented dairy products (Little et al., 2020). We listed the main opportunistic pathogens in different fermented foods and the diseases that may cause diseases in the human body to attract the attention of the fermented food industry and promote the development of the fermented food industry. In addition, antimicrobial resistance is a global threat, and its major determinant is ARGs (Sanderson et al., 2016). Since bacteria can share genetic components by horizontal gene transfer, even non-pathogenic bacteria can provide ARGs to any pathogen that they are physically close to, including in the human intestinal tract (Tan et al., 2021). The consumption of fermented foods increases the risk of transmission of microbial ARGs in the human intestine (Tan et al., 2021). Therefore, we also analyzed the antibiotic resistance genes in different fermented foods, and the results showed that the types and numbers of antibiotic resistance genes in fermented soybean products were the highest (Figure 6F). Our results suggested that the consumption of fermented soy products may be risky.

Taken together, our results uncovered the potential benefits and risks of traditional fermented foods. Fermented dairy products had a high content of beneficial bacteria and a low level of opportunistic pathogens and ARGs, which may make them the most functional fermented foods. However, the risks of consumption were increased due to the enrichment of opportunistic pathogens and ARGs in fermented soy products. Emphatically, potential health promotion attributes were prevalent in fermented foods that benefited from the widespread existence of potential beneficial bacteria, while the potential edible risk of fermented foods should be given more attention. We also analyzed the microbial diversity, taxonomic composition, and metabolic pathways of different fermented foods. In addition, characteristic bacteria were found in different fermented foods, which not only characterized the microbial resources of different fermented foods but also facilitated the development and utilization of microbial resources of fermented foods. The data presented here will provide worthy intelligence or further optimizing the production of fermented foods, utilizing their health promotion potential and reducing their safety risks.

## Data availability statement

The datasets presented in this study can be found in online repositories. The names of the repository/repositories and accession number(s) can be found below: PRJNA890920.

## Author contributions

MX: investigation, project administration, formal analysis, writing—original draft, and data curation. SS, ZZ, SJ, and JZ: project administration, conceptualization, and writing—review and editing. YX and XH: funding acquisition, conceptualization, validation, project administration, and writing—review and editing. All authors contributed to the article and approved the submitted version.
